# First Report of Fruit Rot of Cherry and Its Control Using Fe_2_O_3_ Nanoparticles Synthesized in *Calotropis procera*

**DOI:** 10.3390/molecules27144461

**Published:** 2022-07-12

**Authors:** Mohammad Sameer Zubair, Muhammad Farooq Hussain Munis, Ibtisam M. Alsudays, Khalid H. Alamer, Urooj Haroon, Asif Kamal, Musrat Ali, Junaid Ahmed, Zimen Ahmad, Houneida Attia

**Affiliations:** 1 Department of Plant Sciences, Faculty of Biological Sciences, Quaid-i-Azam University, Islamabad 45320, Pakistan; mszubair@bs.qau.edu.pk (M.S.Z.); uharoonsheikh@gmail.com (U.H.); kamal@bs.qau.edu.pk (A.K.); musrat.ali@bs.qau.edu.pk (M.A.); junaidahmed@bs.qau.edu.pk (J.A.); zimen.ahmad@gmail.com (Z.A.); 2 Department of Biology, College of Science and Arts, Qassim University, Unaizah 56452, Saudi Arabia; absdies@qu.edu.sa; 3 Biological Sciences Department, Faculty of Science and Arts, King Abdulaziz University, Rabigh 21911, Saudi Arabia; kalamer@kau.edu.sa; 4 Department of Biology, College of Science, Taif University, P.O. Box 11099, Taif 21944, Saudi Arabia; hunida.a@tu.edu.sa

**Keywords:** cherry, *Calotropis procera*, Fe_2_O_3_, SEM, *Aspergillus flavus*

## Abstract

Cherry is a fleshy drupe, and it is grown in temperate regions of the world. It is perishable, and several biotic and abiotic factors affect its yield. During April–May 2021, a severe fruit rot of cherry was observed in Swat and adjacent areas. Diseased fruit samples were collected, and the disease-causing pathogen was isolated on PDA. Subsequent morphological, microscopic, and molecular analyses identified the isolated pathogen as *Aspergillus flavus*. For the control of the fruit rot disease of cherry, iron oxide nanoparticles (Fe_2_O_3_ NPs) were synthesized in the leaf extract of *Calotropis procera* and characterized. Fourier transform infrared (FTIR) spectroscopy of synthesized Fe_2_O_3_ NPs showed the presence of capping and stabilizing agents such as alcohols, aldehydes, and halo compounds. X-ray diffraction (XRD) analysis verified the form and size (32 nm) of Fe_2_O_3_ NPs. Scanning electron microscopy (SEM) revealed the spinal-shaped morphology of synthesized Fe_2_O_3_ NPs while X-ray diffraction (EDX) analysis displayed the occurrence of main elements in the samples. After successful preparation and characterization of NPs, their antifungal activity against *A. flavus* was determined by poison technique. Based on in vitro and in vivo antifungal activity analyses, it was observed that 1.0 mg/mL concentration of Fe_2_O_3_ can effectively inhibit the growth of fungal mycelia and decrease the incidence of fruit rot of cherry. The results confirmed ecofriendly fungicidal role of Fe_2_O_3_ and suggested that their large-scale application in the field to replace toxic chemical fungicides.

## 1. Introduction

Cherry belongs to the family *Rosaceae,* and it is a member of the genus Prunus. Cherry fruit has high levels of vital nutrients and bioactive components, including fructose, vitamin C, glucose, anthocyanin, flavonoids, hydroxycinnamate, quercetin, and flavan-3-ols [1]. Carbohydrates (12–17%) and dietary fibers (1.3–2.1%) are the main chemical compounds in cherry. In cherry, the average range of sugar content varies (approximately 11 to 15%), depending upon the climatic conditions, cultivation, and roots system [2]. Cherries provide low calories, and they are rich in vitamins, nutrients, and fiber [3]. 

Cherry is a common crop in Europe and Western Asia, and it is harvested at a large scale, all over the world. Spain, the USA, Chile, and Turkey serve as the main exporters of cherry [3]. It is exported to almost all countries of the world [4]. Cherry is produced in northern areas of Pakistan, on approximately 400 hectares, producing 1660 tons of cherry fruit, annually (FAOSTAT, 2019). Normal and Hunza Valley are the major cherry-producing areas in Pakistan [5]. Other temperate zones for the growth of commercial cherry in Pakistan include Loralai, Pishin, Quetta, Mastung, Kalat, Zhob, and Swat. Recently, cherry has been cultivated in Baluchistan on about 897 hectares, with an annual production of about 1507 tons [6]. 

Many pathogens including bacteria and fungus cause pre-harvest and post-harvest losses of cherry fruit. The devastating diseases of cherry include bacterial canker, X-disease, ripe root rot, brown rot blossom, twig blight, and phytophthora root crown rot [7]. Cherry is a perishable fruit and half of its losses are caused by fungal pathogens [8]. Currently, plant pathogenic fungi are causing more than 50% losses of the total cherry fruit [9]. For the last several decades, many chemical pesticides are being used to control these fungal diseases. Chemical fungicides are frequently quite hazardous, and their direct use on fruits causes numerous human health problems. When the residues of these chemical fungicides accumulate in soil, plants, and animals, they harm other living organisms in the environment and disturb the whole ecosystem [10]. Due to their undegradable nature, these chemical pesticides persist in the system and cause long-term damage. Scientists are thinking to replace these pesticides with some environment-friendly alternatives. Different kinds of bio-fungicides are being introduced in the market to avoid health issues to humans and animals. Bio-fungicides are not toxic and give improved protection to plants. The danger of emerging pathogen resistance can also be reduced by using easily degradable bio-fungicides [11]. Fe_2_O_3_ NPs have been reported to control soft rot of peach [12] and inhibit the growth of *Alternaria alternata* [13].

Nanotechnology is an emerging field to complement other technologies and enhance their performance [14]. For medicinal uses, several nanoparticles are being synthesized and applied [15]. Scientists have begun to produce nanoparticles using plant and other biotic sources. Production of nanoparticles using plant sources is termed as green technology. These green nanoparticles display excellent antifungal properties [16]. Extracts of different medicinal plants are being used to synthesize NPs. Among these medicinal plants, *C. procera* displays important medicinal properties. *C. procera* belongs to the Asclepiadaceae family, and it is found predominantly in Asia and Africa’s tropical and subtropical regions. It includes a variety of bioactive components including polysaccharides, phenolics, flavonoids, terpenoids, and proteins [17]. *C. procera* has been reported to keep powerful antifungal and medicinal properties [18]. 

This study was designed to use *C. procera* for the synthesis of iron oxide nanoparticles (Fe_2_O_3_ NPs). Before the application of Fe_2_O_3_ NPs to control fruit-rot of cherry, they were characterized using sophisticated approaches like Fourier transform infrared (FTIR) spectroscopy, X-ray diffraction (XRD), Scanning electron microscopy (SEM), and Energy-dispersive X-ray (EDX). 

## 2. Results and Discussion

### 2.1. Morphological and Microscopic Identification of the Isolated Pathogen 

**Figure 1:**
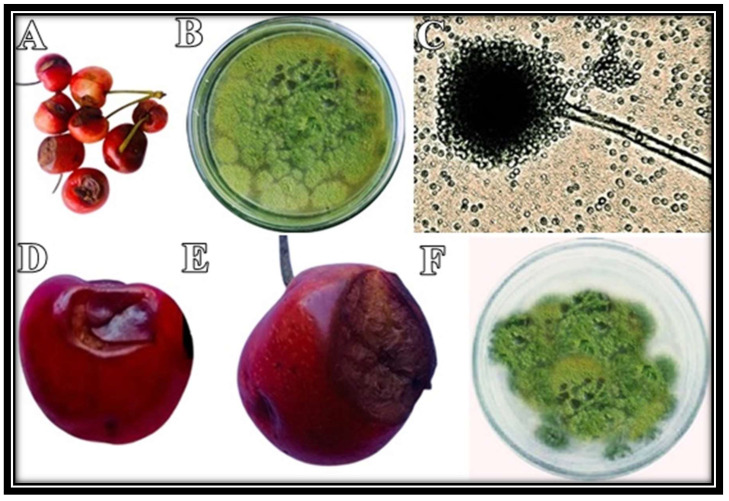
Diseased cherry fruit were collected (**A**), and a disease-causing pathogen was isolated on PDA (**B**). Fungal morphology was observed under microscope (**C**). Following Koch’s postulates, disease symptoms were observed after 3 days (**D**) and 5 days (**E**) post-inoculation. The fungus was re-isolated on PDA (**F**).

Cherry fruit with distinct symptoms were collected from the field (Figure 1A). The isolated pathogen appeared white from the edges and green from the center. Later, greenish colonies covered the entire plate (Figure 1B). Under the microscope, hyaline hyphae with distinct conidial heads and flask-shaped phialides could be observed (Figure 1C). Based on these characteristic features, this fungus was identified as *Aspergillus flavus* [19]. Koch’s postulates successfully confirmed the pathogenicity of the isolated pathogen (Figure 1D–F).

### 2.2. Molecular Identification and Phylogenetic Analysis of the Isolated Pathogen

**Figure 2:**
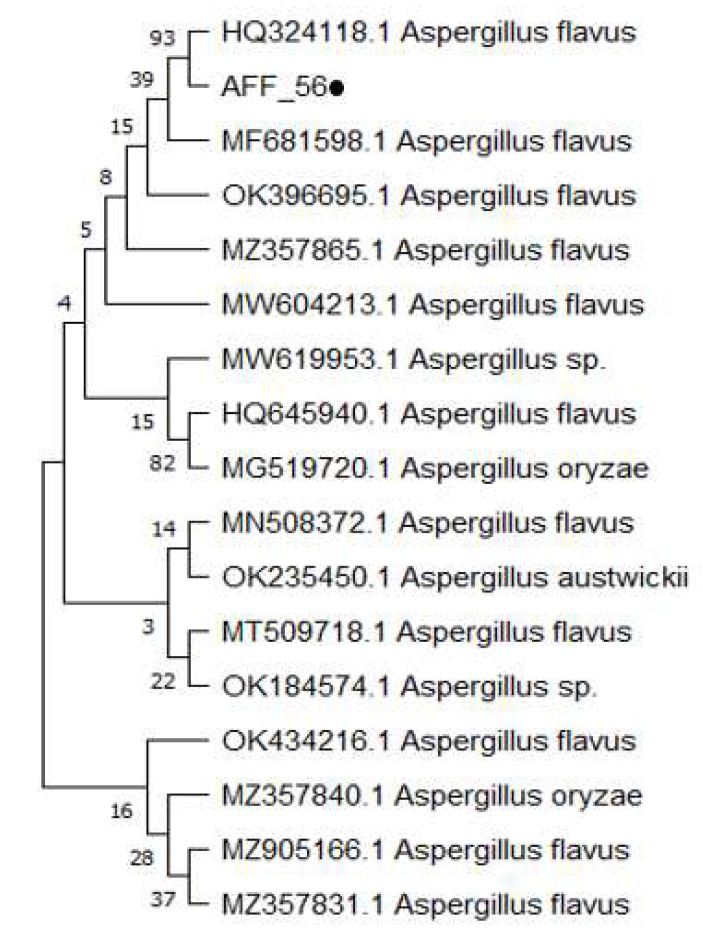
Phylogenetic tree of isolated pathogen.

BLAST analysis of obtained sequence showed 99.63% similarity with *A. flavus* isolate (Accession No. HQ324118.1). The phylogenetic tree also confirmed the evolutionary relationship of isolated fungus with *A. flavus* (Accession No. HQ324118.1) (Figure 2). In the current study, on the basis of morphological, microscopic, and molecular studies, the isolated pathogen was identified as *A. flavus* [20].

### 2.3. Characterization of Nanoparticles

**Figure 3:**
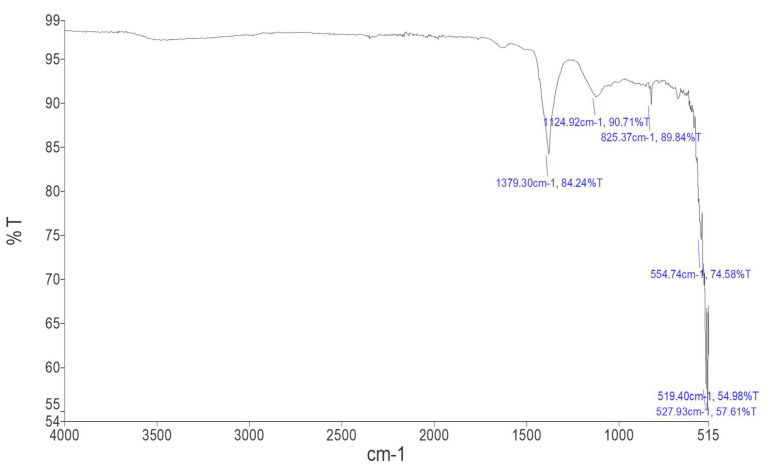
FTIR spectrum of Fe_2_O_3_ NPs synthesized in *C. procera*.

The strongest peak in the FTIR study (Figure 3) was seen in the range from 500 to 1000 cm^−1^, confirming the metal-oxygen bond of Fe_2_O_3_ (N-H). While peaks at 1124.92 cm^−1^ and 825.37 cm^−1^ showed C-O stretching of alcohols and C-Cl stretching of halo compounds, respectively, the absorption band at 1379 cm^−1^ showed C-H bending of surface adsorbed water molecules. The presence of peak at 554.74 cm^−1^ indicates C-Br stretching. The multiple strong peaks at 517.40 cm^−1^ and 527.93 cm^−1^ represented the halo chemical groups. The above-described peaks visibly depict the occurrence of protein on Fe_2_O_3_ NPs. Proteinaceous components of the leaf extract likely act as a capping and stabilizing agent [21]. Protein residues bind to the surface of metal nanoparticles, in the presence of free carboxylate ions or amine groups [22]. In this study, the nanoparticles were characterized by FTIR analysis and confirmed the presence of aldehyde, alcohols, and halo compounds. The results clearly describe the occurrence of protein on the surface of iron nanoparticles. The proteinaceous component of the leaf extract is thought to serve as a stabilizing and capping agent [20]. Different compounds present on the surface of Fe_2_O_3_ NPs that improved their stability are shown in (Table 1).

**Figure 4:**
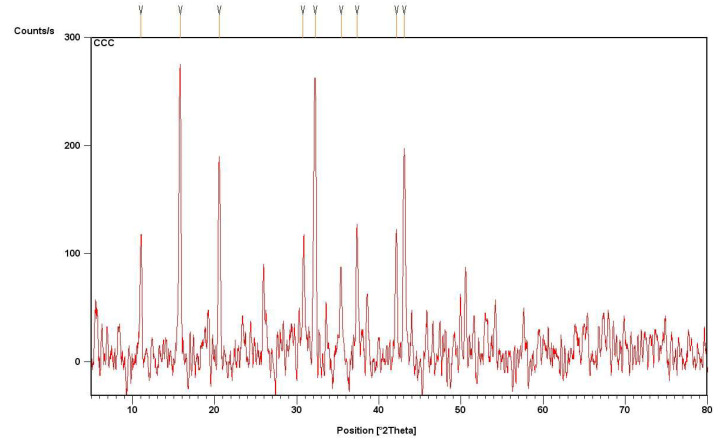
XRD analysis of Fe_2_O_3_ NPs synthesized in *C. procera*.

The XRD pattern dsplayed distinct diffraction peaks of Fe_2_O_3_ NPs (Figure 4). XRD spectra displayed clear peaks 2θ, corresponding to Orthorhombic with Pmc2 space group, representing magnetite iron oxide. The XRD plane patterns are in decent agreement with JSCPD number 01076-0958. The average nanoparticle size was determined to be 32 nm, which was calculated by Scherrer equation; the results were presented in Table 2. XRD alignment for Fe_2_O_3_ NPs is in agreement with previous studies [23]. Iron oxide nanoparticles were extremely crystalline in form, as indicated by the sharp and intense peaks. The purity of prepared nanoparticles was not indicated by any other distinguishing peak. 

**Figure 5:**
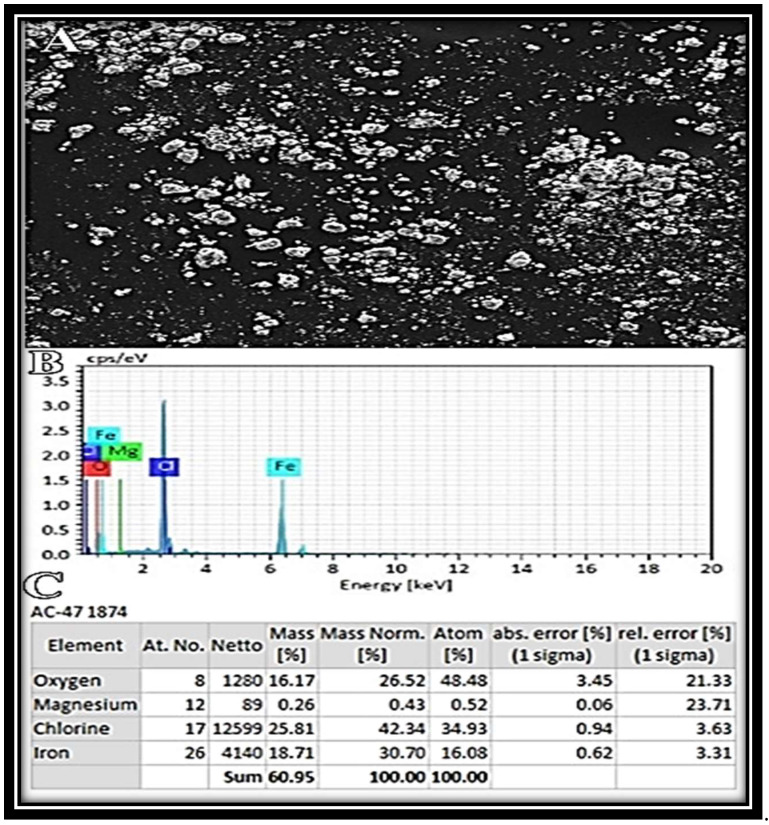
SEM image of Fe_2_O_3_ NPs (**A**). EDX spectrum (**B**) and its values (**C**).

The morphology of Fe_2_O_3_ NPs was successfully revealed with a scanning electron microscope (Figure 5A). Synthesized NPs were uneven in appearance. EDX determined the elemental makeup of Fe_2_O_3_ NPs (Figure 5B). The overall weight percentages of oxygen, chlorine, and iron in prepared Fe_2_O_3_ NPs were 16.17%, 25.81%, and 18.71%, respectively (Figure 5C).

**Figure 6:**
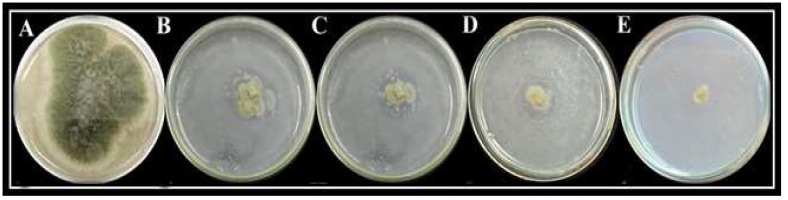
Growth inhibition of *A. flavus* at different concentration of Fe_2_O_3_ NPs. Control (**A**), 0.25 mg/mL concentration of Fe_2_O_3_ NPs (**B**), 0.50 mg/mL concentration (**C**), 0.75 mg/mL concentration (**D**), and 1.0 mg/mL concentration (**E**).

Different concentrations of Fe_2_O_3_ NPs exhibited a variable inhibitory effect on mycelial growth of *A. flavus* (Figure 6). The maximum mycelial growth inhibition (89.6%) was observed at 1.0 mg/mL concentration of Fe_2_O_3_ NPs (Table 2).

### 2.4. Disease Control of Fe_2_O_3_ NPs, In Vitro and In Vivo

Different concentrations of Fe_2_O_3_ NPs exhibited a variable inhibitory effect on mycelial growth of *A. flavus* (Figure 6). The maximum mycelial growth inhibition (89.6%) was observed at 1.0 mg/mL concentration of Fe_2_O_3_ NPs (Table 3).

**Figure 7:**
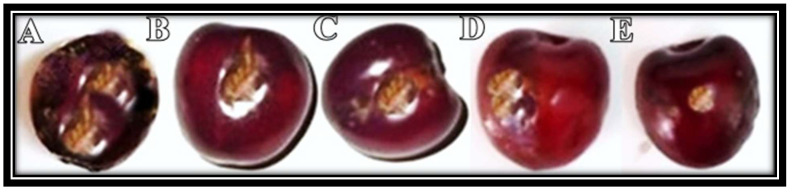
Application of Fe_2_O_3_ NPs to control disease incidence on the cherry. Maximum disease incidence was observed in the control fruit (**A**). Variable disease incidence was observed at 0.25 mg/mL concentration (**B**), 0.50 mg/mL concentration (**C**), 0.75 mg/mL concentration (**D**), and 1.0 mg/mL concentration (**E**).

Variable disease incidence was observed after 5 days of inoculation (Figure 7). All nano-fungicides decreased disease incidence, and the maximum disease control was observed at 1.0 mg/mL concentration of Fe_2_O_3_ NPs (Table 4).

In the current study, different concentrations of Fe_2_O_3_ NPs facilitated *C. procera* leaf extract to control the growth of *A. flavus*. It is reported that the antimicrobial properties of NPs extremely depend upon the crystal-like nature and the size of NPs [24]. Crystalline NPs damage the cell wall of the fungus [25]. Iron oxide nanoparticles have a small size and a large surface-to-volume ratio, so they can strongly adhere to the fungal cell surface. They can also directly enter the cell and cause damage to the cell wall. Fungal inactivation by Fe_2_O_3_ NPs includes a direct contact between NPs and cell surfaces, which causes oxidative stress in fungal cells, resulting in cell death [26]. Fe_2_O_3_ NPs after attachment, rupture membrane and they can also enter through nano-pores [27]. Due to minor size, NPs can penetrate microbial membrane [28]. The incidence of this disease was successfully controlled by Fe_2_O_3_ NPs synthesized in *C. procera.* Control of fruit rot with biotic fungicides is an area of great interest. In the civilized world, the use of chemical pesticides is being discouraged for better human health. Scientists are concentrating on using antimicrobial products, particularly of plant origin, because of their effectiveness and no harmful side effects. In this study, green (plant mediated) synthesis of nanoparticles has been optimized. For the large-scale development of biocompatible and biodegradable nanoparticles, it has become a well-known biomimetic method. Because the major raw material (plants) is typically available naturally and abundantly, the green synthesis of nanoparticles is a cost-effective method [20].

## 3. Materials and Methods

### 3.1. Collection of Rotten Cherry Fruits

Cherry bearing fruit-rot symptoms were collected from Matta Khararai, Swat, Khyber Pakhtunkhwa (34°56′9″ N 72°24′12″ E). Collected samples were placed in polythene bags and transferred to Molecular Plant Pathology Lab, Quaid-i-Azam University, Islamabad, Pakistan.

### 3.2. Isolation of Pathogen

Surface of collected diseased cherry fruits was sterilized with 70% ethanol and diseased parts (3 mm diameter) were excised using sterilized scalpels. These infected fruit segments were plated on solidified Potato Dextrose Agar (PDA) plates, aseptically. Inoculated plates were incubated at 25 ± 2 °C for 5–6 days. After emergence, fungal colonies were sub-cultured to obtain a pure culture of the isolated pathogen.

### 3.3. Macroscopic and Microscopic Identification of Isolated Pathogen

Isolated pathogen was observed on Petri plates after one week, and their mycelial features were recorded. Microscopy of the isolated pathogen was also performed, using the slide culture technique to observe cultural and morphological features such as colony color, conidial morphology, and colony development. For this purpose, an authentic protocol was followed [29]; a drop of lactic acid was placed on a glass slide. Using a sterilized inoculating loop, a small portion of mycelia from the purified culture was taken and placed on the slide. Lactophenol blue was applied on mycelia, and a coverslip was placed carefully to prevent any air bubbles. Slides were allowed to dry and viewed under a light microscope at 100× magnification. The physical traits and shape of the disease-causing organism were validated with the aid of the Mycological Atlas of Robert and Ellen [30].

### 3.4. Molecular Identification of Fungal Species

Fungal DNA was extracted from the mycelia of the one-week-old culture, using CTAB method [31]. The ribosomal internal transcribed spacer (ITS) region was amplified utilizing ITS forward (ITS1) and reverse primer (ITS4). Amplified PCR product was sequenced, and the similarity of the obtained sequence was determined on NCBI GenBank using BLAST tool. A phylogenetic tree was constructed using MEGA software (Molecular Evolutionary Genetics Analysis, Version 7.0.21).

### 3.5. Pathogenicity Test

Freshly collected, fully developed, and unripened fruit were surface sterilized with 70% alcohol and washed with distilled water. Using a sterilized cork borer, holes of 3 mm diameter were produced in healthy fruit. Discs were taken from 7-day old agar culture plates and positioned in the holes. The holes were completely sealed with Vaseline, and the fruit were covered with a muslin cloth. All fruit were incubated at 25 ± 2 °C for 5 days. After disease incidence, PDA media was used to isolate pathogen. 

### 3.6. Collection and Preparation of Leaf Extract of Calotropis procera

Leaves of *C. procera* were collected from various localities of Islamabad and Rawalpindi and washed properly to clean dust particles. These leaves were dried in shade and crushed into fine powder. The powder was mixed in 500 mL water in a flask and boiled for 30 min. The extract was filtered by using a muslin cloth and Whatman filter paper. Pure leaf extract was stored at 4 °C.

### 3.7. Synthesis of Iron Oxide Nanoparticles (Fe_2_O_3_ NPs)

For the preparation of Fe_2_O_3_ NPs, 1 Mm salt of iron acetate hexahydrate was mixed with the extract of *C. procera* in 1:2 ratios. The stirring of mixture was performed on hot plate at 70 °C for 2 h, until the color of Fe_2_O_3_ NPs turned blackish, to confirm salt reduction. The mixture was calcinated and subjected to further characterization.

### 3.8. Characterization of Nanoparticles

Before their further application to control fruit rot disease, synthesized Fe_2_O_3_ NPs were characterized by the following sophisticated approaches.

### 3.9. Fourier Transformed Infrared (FTIR) Spectroscopy

FTIR spectroscopy was used to detect functional groups present on the surface of Fe_2_O_3_ NPs. For this purpose, the standard KBr pellet technique was used. Using a Bruker, Vertex 70 FTIR spectrometer, the synthesized nanoparticles were crushed and merged with 150 mg KBr. Foe analysis, the spectrum range of 4000 cm^−1^ to 400 cm^−1^, was used.

### 3.10. X-ray Diffraction Analysis of Iron Oxide Nanoparticles

To determine the size and the crystalline nature of Fe_2_O_3_ NPs, XRD analysis was performed. X’Pert High Score software determined crystalline features, and the size of nanoparticles by the below formula (Scherrer Equation (1)):

D = kλ/βcosθ(1)

where D = average crystalline size perpendicular to the reflecting planes, K = shape factor, λ = X-ray wavelength, β = full width at the half maximum (FWHM), θ = diffraction angle.

### 3.11. Scanning Electron Microscopy (SEM) and Energy Dispersive X-ray (EDX) Analysis

A sonicated Fe_2_O_3_ NP solution was produced in double-distilled water. On double carbon coating conductive tape, a droplet of the sonicated solution was applied and dried using a lamp. SEM and EDX analyses were performed using ionic emission SEM equipment (VEGA3 TESCAN). 

### 3.12. Application of Fe_2_O_3_ NPs, In Vitro

Mycelial growth inhibition of isolated fungus was determined at different concentrations of Fe_2_O_3_ NP, using the poisoned food technique. PDA media were amended with four different concentrations (1.0 mg/mL, 0.75 mg/mL, 0.5 mg/mL, and 0.25 mg/mL) of Fe_2_O_3_ NPs and dispensed into Petri plates. In the center of solidified PDA media plate, a fungal disc of 5 mm diameter was placed. PDA media without Fe_2_O_3_ NPs served as the control. The whole experiment was performed in three replicates. All Petri plates were incubated at 25 ± 2 °C. After 7 days of incubation, mycelial growth inhibition was measured by the following formula Equation (2):

Growth inhibition % = C − T/C × 100(2)

where, C = average mycelial growth in positive control Petri plates; T = average mycelial growth in treated Petri plates. 

### 3.13. Application of Fe_2_O_3_ NPs to Control Fruit Rot, In Vivo

In an in vivo experiment, cherry fruit were inoculated with fungus (like a pathogenicity test) and placed in an incubator at 25 ± 2 °C. After two days of incubation, inoculated fruit were sprayed (till run off) with different concentrations (1.0 mg/mL, 0.75 mg/mL, 0.5 mg/mL, and 0.25 mg/mL) of Fe_2_O_3_ NPs and placed again at 25 ± 2 °C. Infected fruit were sprayed again with Fe_2_O_3_ NPs on the fourth day post-inoculation. The control infected cherry fruit were sprayed with distilled water. The diseased area was calculated after one week of inoculation.

### 3.14. Statistical Analysis

The experiment was conducted in three replicates unless otherwise stated. Mean and standard deviation was obtained using MS-Excel 2016 software (Microsoft Inc., Redmond, WA, USA).

## 4. Conclusions

To the best of our knowledge, it is the first study that treated the fruit rot disease of cherry by isolating and identifying its causal organism. The isolated pathogen (*A. flavus*) was controlled by iron oxide NPs prepared in the leaf extract of *C. procera*. The findings of this study demonstrated that the bioactive components found in the leaf extract of *C. procera* effectively reduce and stabilize iron oxide nanoparticles. The growth of *A. flavus* can be greatly slowed down by small iron oxide nanoparticles. They can be used on fruits and vegetables as these nanoparticles are also environmentally friendly and economically cheaper. For a sustainable environment, these green nanoparticles (bio-pesticides) should replace harmful chemical pesticides in the field.

## Figures and Tables

**Table 1:** FTIR spectral values of Fe_2_O_3_ NPs synthesized in *C. procera*.

Peak Number	Absorption (cm^−1^) in Sample	Absorption (cm^−1^) in Standard Table	Appearance	Group	Compound Class
1	1379.30	1390–1380	Medium	C-H bending	Aldehyde
2	1124.92	1124–1087	Strong	C-O stretching	secondary alcohol
3	825.37	850–550	Strong	C-Cl stretching	halo compound
4	554.74	690–515	Strong	C-Br stretching	halo compound
5	517.40	600–500	Strong	C-I stretching	halo compound
6	527.93	690–515	Strong	C-Br stretching	halo compound

**Table 2:** Size of Fe_2_O_3_ synthesized using leaf extract of *C. procera*.

Peak Number	2θ	D (nm)	Average Size
1	11	30.432	32.261
2	15.5	29.143	
3	20.8	25.678	
4	32	23.511	
5	43.5	24.806	
6	50.7	59.995	

**Table 3:** Growth inhibition of *A. flavus* at *concentration of* Fe_2_O_3_ NPs.

Treatment	Growth Inhibition (%)
0.25 mg/mL	55.8 ± 2.1
0.50 mg/mL	62 ± 4.6
0.75 mg/mL	75.8 ± 4.1
1.0 mg/mL	89.6 ± 2.2

**Table 4:** Diseased area of cherry fruit under different concentrations of Fe_2_O_3_ NPs.

Treatment	Diseased Area (mm)
Control	81 ± 2.1
0.25 mg/mL	39.8 ± 2.1
0.50 mg/mL	34 ± 4.6
0.75 mg/mL	30.8 ± 4.1
1.0 mg/mL	25 ± 2.2

## Data Availability

The datasets generated during and/or analyzed during the current study are available from the corresponding author on reasonable request.

## Sample Availability

No samples have been produced for public.
